# Evolving Data‐Driven Strategies for the Characterization of Supramolecular Polymers and Systems

**DOI:** 10.1002/anie.202509122

**Published:** 2025-08-22

**Authors:** Stef A. H. Jansen, Ghislaine Vantomme, E. W. Meijer

**Affiliations:** ^1^ Institute for Complex Molecular Systems and Laboratory of Macromolecular and Organic Chemistry Eindhoven University of Technology P.O. Box 513, 5600 MB Eindhoven The Netherlands; ^2^ School of Chemistry and RNA Institute, UNSW Sydney Australia; ^3^ Max Planck Institute for Polymer Research Ackermannweg 10 55128 Mainz Germany

## Abstract

Inspired by the dynamic assembly of fibrillar proteins in biology, research on supramolecular polymers has progressed rapidly toward the development of synthetic multicomponent systems. In this review, we highlight recent advances in the study of supramolecular polymers in solution, with an emphasis on how combined computational and experimental approaches deepen our understanding of these systems. In particular, these studies have elucidated the mechanisms of protein aggregation and provided insights into the characteristics of synthetic systems. We discuss the classification of these polymers and systems, highlighting how their different interaction modes and microstructures give rise to diverse structural and functional properties. In addition, we outline the emerging role of machine learning as a powerful tool to navigate the inherent complexity of these systems, thereby enhancing strategies for rational design and characterization. This review highlights how computational approaches, from traditional modeling to emerging machine learning techniques, enable the experimental characterization and understanding of supramolecular polymer chemistry.

## Graphical abstract

Research on supramolecular polymers has advanced rapidly toward the development of synthetic multicomponent systems. This review highlights recent progress in understanding their assembly mechanisms and how these influence structural and functional properties. Emerging data‐driven approaches, particularly machine learning, offer new opportunities for rational design and characterization, shaping the future of supramolecular chemistry.

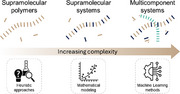

## 1 Introduction

The dynamic structural properties of fibrillar protein assemblies are essential for cell function, making them ubiquitous in nature.^[^ ^1^ ^]^ The protein filaments of the cytoskeleton, such as actin, microtubules, and intermediate filaments, form dynamic networks that support intracellular transport,^[^ ^2^ ^]^ signal transduction,^[^ ^3^ ^]^ and force generation.^[^ ^4^ ^]^ Meanwhile, in the extracellular environment, supramolecular assemblies such as collagen fibrils or elastin provide the mechanical framework that influences cell morphology, proliferation, and differentiation.^[^ ^5^ ^]^ The formation of these supramolecular assemblies is driven by non‐covalent interactions, such as hydrogen bonding, van der Waals forces, and electrostatic interactions between protein subunits. The reversible nature of these interactions yields inherently dynamic assemblies, capable of responding to a variety of stimuli such as changes in pH, temperature, and ionic strength, which allows cells and tissues to adapt to different physiological conditions. However, this dynamicity also introduces a degree of sensitivity, as small perturbations can cause protein misfolding and subsequent formation of dysfunctional, toxic aggregates. This process is implicated in a wide range of pathological conditions, including neurodegenerative diseases such as Alzheimer's, Parkinson's, and Huntington's.^[^ ^6^ ^]^ Understanding the mechanisms underlying the assembly and disassembly of protein aggregates is therefore a major focus of research aimed at developing therapeutic strategies for protein misfolding diseases.^[^ ^7^ ^]^

Inspired by natural supramolecular assemblies, exploiting non‐covalent interactions between small molecules in synthetic supramolecular polymers^[^ ^8^, ^9^ ^]^ has led to exciting achievements in biomaterials and optoelectronics.^[^ ^10^ ^]^ Supramolecular polymer chemistry is now a thriving field, bridging the disciplines of synthetic organic chemistry, polymer chemistry, and materials science. The development of supramolecular polymer applications is driven by answering fundamental questions about the underlying mechanisms of assembly. Which principles govern the assembly of synthetic and natural supramolecular polymers? How can the stability of supramolecular polymers be controlled? Which factors determine the size of supramolecular assemblies? What are the similarities and difference between synthetic and natural supramolecular polymers? Answers to these and many other questions have been found in theoretical frameworks that describe supramolecular polymerizations under kinetic or thermodynamic control. Especially when combined with experiments, each of these studies advances the understanding of supramolecular systems and also raises more fundamental questions, and ultimately bringing us one step closer to developing supramolecular polymer technologies for positive societal impact.

Despite these efforts, synthetic supramolecular chemistry is far from matching the intricacy of its natural counterpart. The emergent properties and functionalities of complex systems motivate to address their inherent sensitivity and issues with reproducibility.^[^ ^11^ ^]^ However, synthetic supramolecular polymers have mostly been studied as isolated structures dispersed in a solvent. Only in recent years, researchers have started to explore increasingly complex environments and multiple interacting components in the field of supramolecular systems and materials.^[^ ^12^ ^]^

Here, we review the progress made in the characterization of supramolecular polymers and systems. We reflect on the computational methods developed to support the experimental study of protein aggregation and their translation and application to the synthetic counterparts. Finally, we present the first examples of data‐driven approaches applied to the understanding and design of supramolecular polymers, underscoring the significant potential of machine learning (ML) to simplify and accelerate the exploration of complex multicomponent systems.

## 2 Supramolecular Protein Assemblies

**Figure 1:**
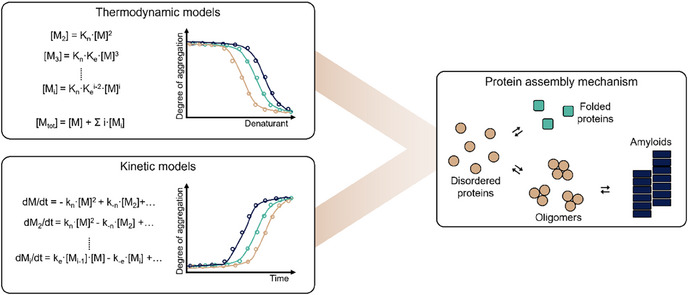
Illustrative overview of modeling methods used to elucidate protein assembly mechanisms. Thermodynamic models based on mass‐balance equations (simplified in the overview) can be fitted to data from denaturation experiments or other experiments of protein assembly at thermodynamic equilibrium. Kinetic models based on ordinary differential equations (simplified in the overview) can be fitted to kinetic experimental traces of protein assembly. The assembly mechanisms included in the models can be adjusted to find the best fit, providing insight into the possible assembly mechanisms for an experimental system.

Extensive studies, pioneered by Prof. Chris Dobson at the University of Cambridge, have been conducted to elucidate the principles governing the formation of supramolecular protein assemblies, and thereby identify new targets for therapeutics to treat protein misfolding diseases.^[^ ^6^ ^]^ Computational methods have become indispensable tools in this endeavor,^[^ ^13^ ^]^ allowing researchers to simulate the kinetics and thermodynamics of protein assembly, providing insight into the molecular mechanisms that drive these processes (Figure 1).^[^ ^14^ ^]^ In particular, mass‐balance modeling allows the detailed study of protein assembly processes and the prediction of assembly equilibria under varying conditions.^[^ ^15^ ^]^ The core of this approach is a set of mathematical equations that describe the concentrations of free and assembled subunits in terms of their respective binding affinities and stoichiometries.^[^ ^16^ ^]^ By solving these equations, it is possible to predict how changes in environmental conditions, such as protein concentration or temperature, will affect the stability and size of the supramolecular assembly under thermodynamic control. On the other hand, kinetic modeling has been successfully used to reveal mechanistic information and pathways of protein aggregation.^[^ ^17^ ^]^ For example, the aggregation mechanism of sickle hemoglobin,^[^ ^18^, ^19^ ^]^ actin filaments,^[^ ^14^, ^20^ ^]^ glutamate dehydrogenase,^[^ ^21^, ^22^ ^]^ microtubules,^[^ ^23^ ^]^ and pathways for amyloid formation^[^ ^24^, ^25^ ^]^ have been determined using thermodynamic and kinetic modeling. Collectively, these studies demonstrate that mathematical models are powerful tools for the fundamental study of protein assembly.

In addition to advancing the mechanistic understanding of protein aggregation, these computational methods offer the ability to design experiments more efficiently than would be possible using traditional laboratory techniques alone. By fitting models to experimental data, the obtained fitting parameters can contain information about the physical properties of the system,^[^ ^17^ ^]^ and interpolation of the experimental data can yield the predicted response of the system in unperformed experiments. In this way, the modeling approaches can be used to simulate how small molecules or drugs affect protein assembly dynamics, providing a framework for drug discovery aimed at stabilizing or disrupting specific supramolecular structures.^[^ ^26^ ^]^ This is particularly important in the context of developing therapeutics for protein misfolding diseases, where the goal is often to either inhibit the aggregation of misfolded proteins or promote the clearance of existing aggregates.^[^ ^27^ ^]^ By integrating experimental data with theoretical models, the understanding of the molecular mechanisms that govern protein assembly can be refined, and more targeted approaches can be developed to treat diseases associated with protein aggregation.

## 3 Synthetic Supramolecular Polymers

**Figure 2:**
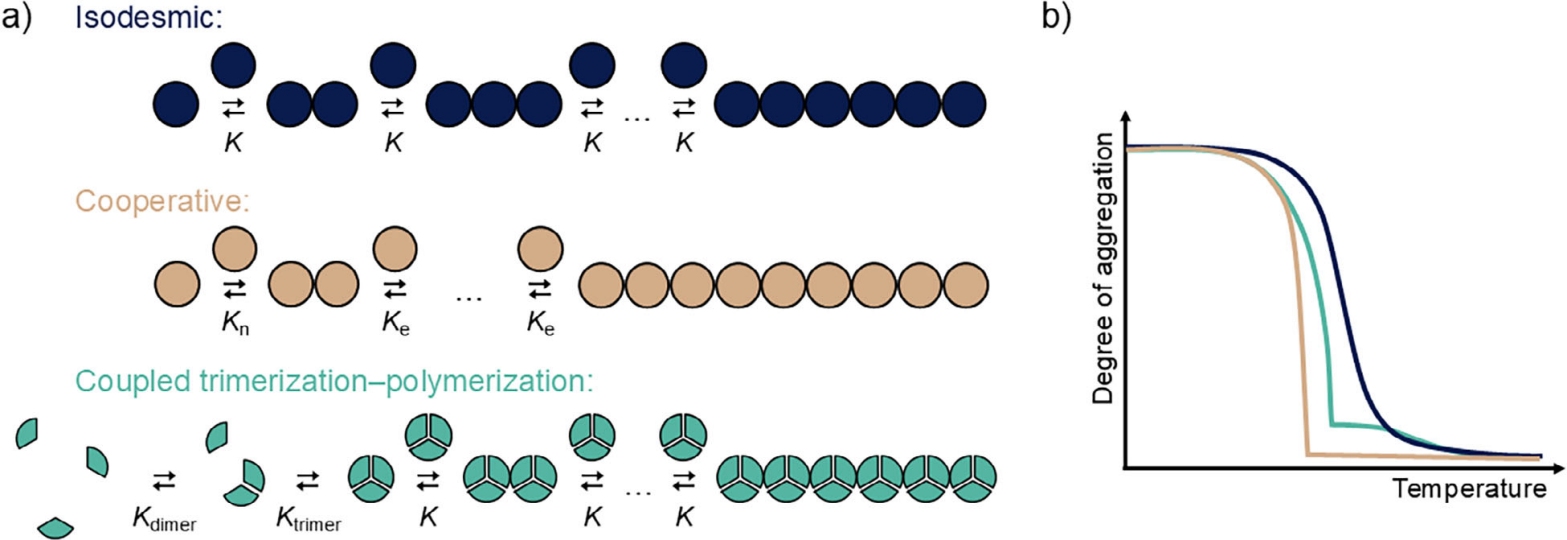
a) Schematic representation of isodesmic and cooperative supramolecular polymerization mechanisms, and an exemplary mechanism for coupled trimerization–polymerization equilibria, b) Schematic representation of typical variable temperature spectroscopic experiments for the assembly mechanisms in a).

Inspired by the wide variety of structures and functions of supramolecular polymers in nature, synthetic polymers based on directional non‐covalent interactions were first reported in 1988 by Aida and co‐workers.^[^ ^8^ ^]^ In 1997, the development of the first mechanically stable supramolecular polymer with mechanical properties competitive with covalent polymers marked the birth of the field of synthetic supramolecular polymer chemistry.^[^ ^9^ ^]^ Since then, supramolecular polymers for aqueous environments have been developed, such as peptide amphiphiles pioneered by Stupp and co‐workers,^[^ ^28^ ^]^ which opened the door to biomedical applications by mimicking natural supramolecular assemblies. Extensive research has been directed toward understanding supramolecular polymerization and leveraging this knowledge to gain precise control over the assembly process.^[^ ^29^ ^]^ The study of synthetic supramolecular polymers involves many of the same characterization methods and theoretical concepts used for natural assemblies. For example, the classification of protein assemblies based on their formation mechanisms (isodesmic polymerizations, where each binding event has the same binding constant, and cooperative polymerizations, where the formation of a relatively unfavorable nucleus is followed by elongation with higher binding constants, Figure 2) has been transferred to supramolecular polymerizations and has become part of the standard terminology.^[^ ^30^ ^]^

Due to the distinct mechanism, cooperative polymerizations can typically form long polymer chains compared to isodesmic polymerizations.^[^ ^31^, ^32^ ^]^ The equilibrium constants of supramolecular polymerizations can be calculated using Van ‘t Hoff analysis, which yields the entropy and enthalpy change upon aggregation by applying linear regression to experimental data.^[^ ^33^ ^]^ However, the quantification of cooperativity requires the fitting of thermodynamic models, which have been introduced previously for the mathematical description of protein aggregation.

**Coupled Trimerization and Polymerization Equilibria**

**Figure 3:**
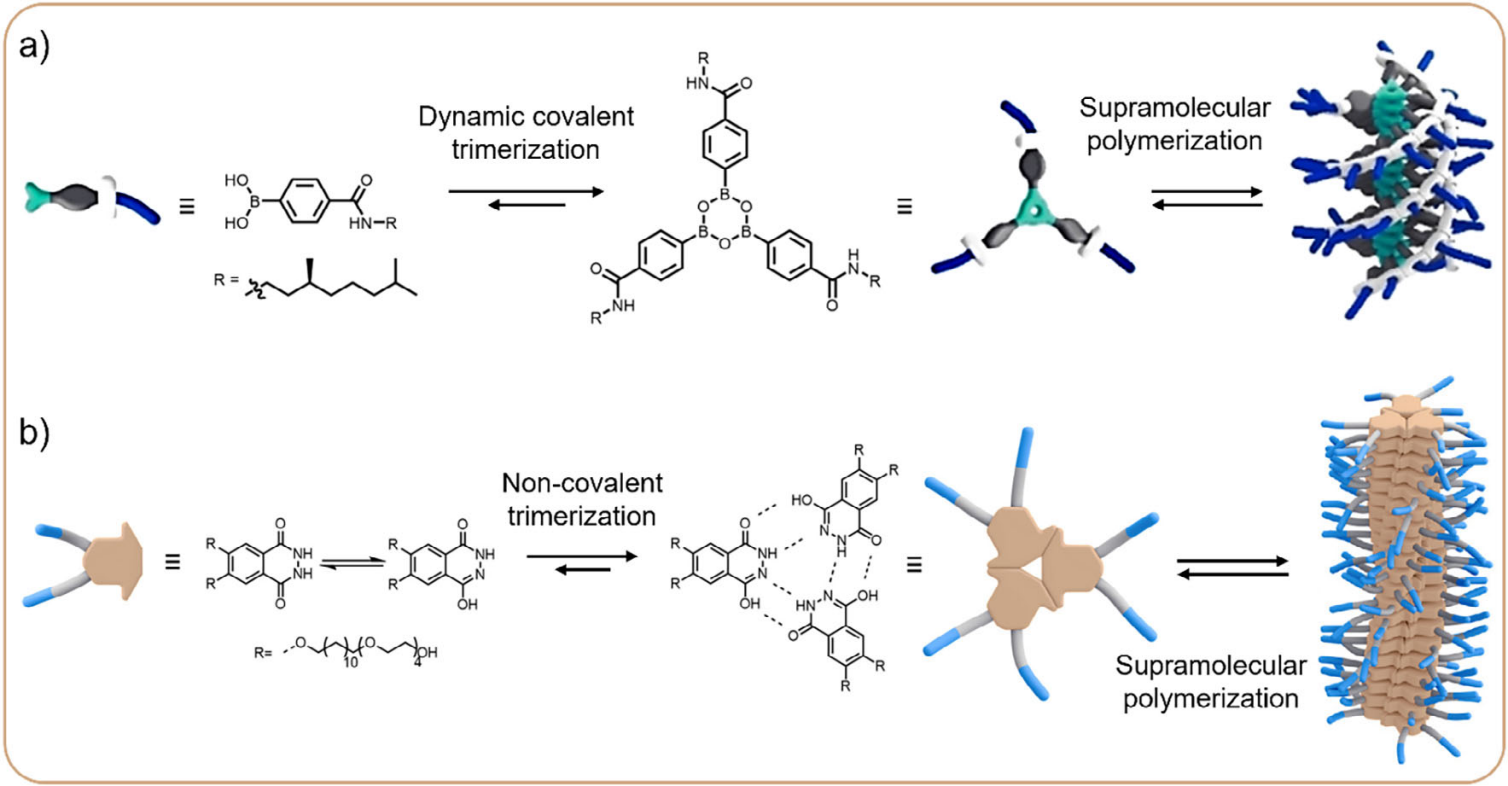
Molecular structures and schematic representation of a) dynamic covalent trimerization of boronic acids to form boroxines, which subsequently stack into elongated supramolecular polymers in organic solvents. Adapted with permission from Ref.[34], © 2024, Wiley‐VCH GmbH. b) Non‐covalent trimerization of phthalhydrazide monomers into trimeric disks that stack into supramolecular polymers in water. Adapted with permission from Ref.[35], © 2024, Chinese Chemical Society.

Many monomers exist that do not assemble co‐facially into one‐dimensional aggregates, but rather first form small oligomers that then act as the repeating units in the directional supramolecular polymer assemblies (Figure 2, in cyan). For example, we have experimentally studied boronic acids in organic solvents that covalently trimerized into boroxine monomers before stacking into larger structures (Figure 3a).^[^ ^34^ ^]^ The dynamic covalent trimerization equilibrium could be quantitatively shifted to the boroxines by removing the water that is formed as a byproduct. The coupled equilibria in this system allowed for easy functionalization of the polymers by mixing in boronic acids with appended functional groups in the desired amount. Similarly, we have studied phthalhydrazide units in water, of which the lactim‐lactam tautomer formed non‐covalent trimers that assembled into elongated aggregates (Figure 3b).^[^ ^35^ ^]^ In this case, the coupled trimerization–polymerization equilibria resulted in cooperative assembly, which is uncommon for water‐soluble supramolecular polymers. Both supramolecular polymerization mechanisms were successfully characterized using mass‐balance models to extract the thermodynamic stabilities of the assembled species, highlighting the versatility of this method.

**Figure 4:**
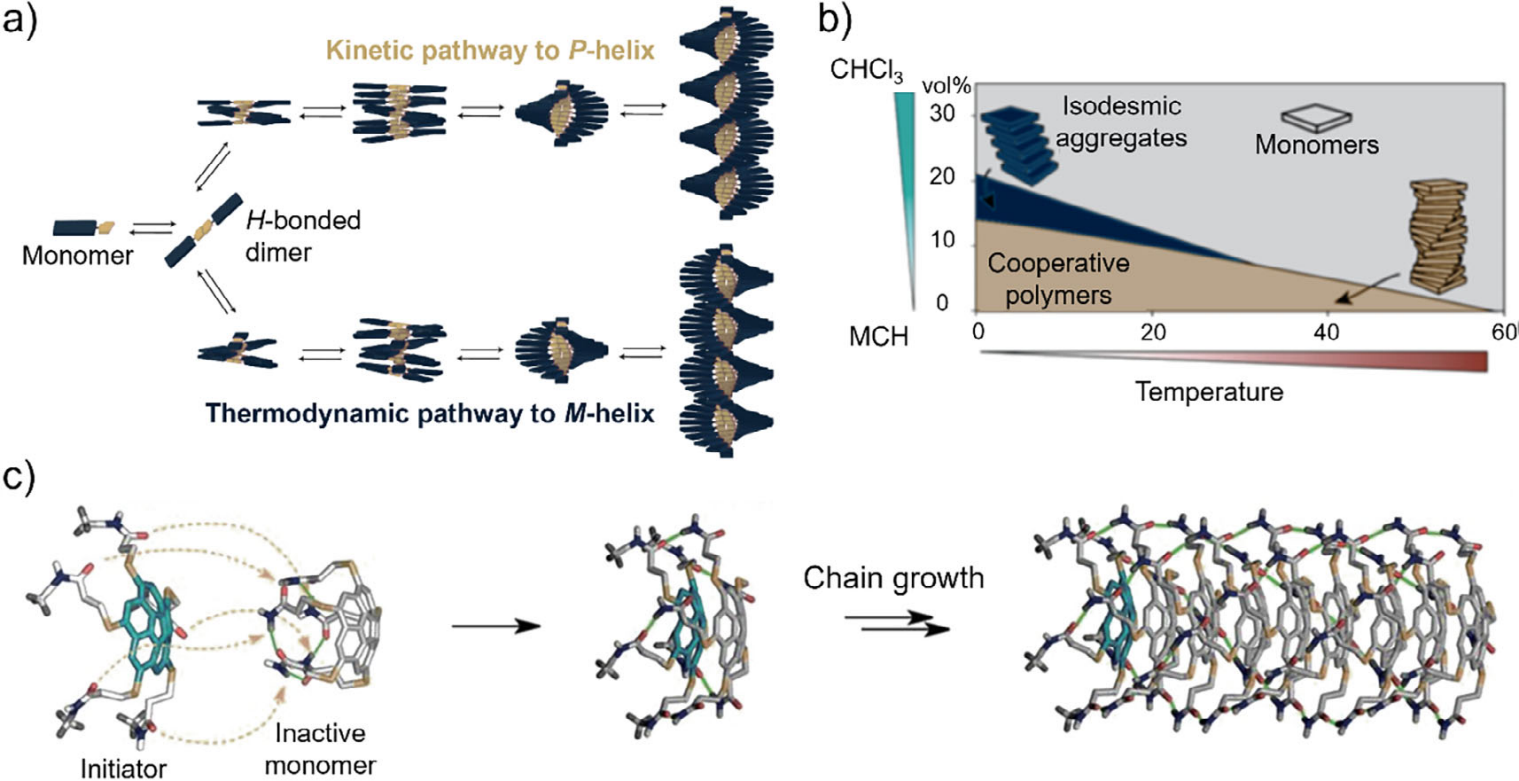
a) Pathway complexity, illustrated by an example of SOPV monomer aggregation into P‐ and M‐helices. Adapted with permission from Ref.[38], © 2012, Springer Nature Limited. b) Schematic phase diagram of pathway complexity in Zn‐porphyrin assembly into H‐ and J‐type aggregates as a function of temperature and solvent composition. c) Schematic illustration of a living supramolecular polymerization mechanism triggered by the addition of initiator to an intramolecularly hydrogen‐bonded monomer. Adapted with permission from Ref.[49], © 2015, The American Association for the Advancement of Science.

Synthetic supramolecular polymers have traditionally been studied at or near thermodynamic equilibrium. However, many supramolecular polymerizations do not occur strictly at thermodynamic equilibrium. Kinetically preferred structures are often formed, which can lead to competing pathways to different final assembly structures, as is common in protein assemblies.^[^ ^36^, ^37^ ^]^ This principle of pathway complexity can be exploited to achieve more diverse structures of synthetic polymers, but often poses a challenge in their design.^[^ ^38^, ^39^ ^]^ Mathematical models have been successfully applied to elucidate pathway complexity.^[^ ^40^ ^]^ For example, kinetic models were used to rationalize the competition between *P*‐ and *M*‐helical aggregates of chiral oligo(*p*‐phenylenevinylene) monomers (Figure 4a),^[^ ^38^ ^]^ and thermodynamic models were used to elucidate the competition between *H*‐ and *J*‐type aggregates for different porphyrin monomers (Figure 4b).^[^ ^41^, ^42^ ^]^ These analyses revealed the precise conditions that must be adjusted to direct the supramolecular assembly toward the desired aggregate type.

By making use of pathway complexity, non‐equilibrium supramolecular polymerizations can be achieved.^[^ ^43^ ^]^ Kinetic assembly products can be obtained by selecting the assembly pathway, for example using solvent processing methods^[^ ^44^, ^45^ ^]^ or performing stepwise assembly.^[^ ^46^, ^47^ ^]^ A special case of non‐equilibrium assembly is the seeded “living” supramolecular polymerization, where the chain growth can be initiated and controlled by adding nuclei for the polymerization (Figure 4c).^[^ ^48^ ^]^ In a seminal report by Aida and co‐workers, living supramolecular polymerization of intramolecularly hydrogen‐bonded monomers was induced by addition of an initiator monomer.^[^ ^49^ ^]^ The rate constants for (dis)assembly in living supramolecular polymerizations was quantified with a seeded polymerization kinetics model.^[^ ^50^ ^]^

**In Situ Synthesis of Supramolecular Polymers**

**Figure 5:**
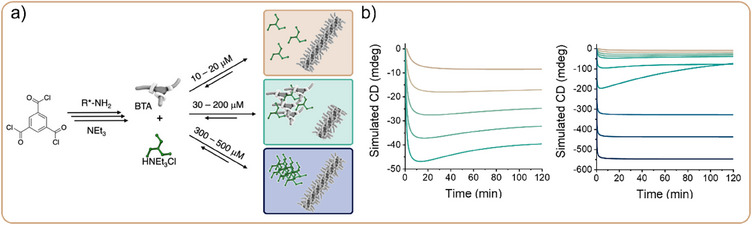
a) Schematic overview of the in situ synthesis of BTA supramolecular polymers. The triacid chloride is substituted by the amine side chain to form BTA in methylcyclohexane, which promotes assembly into supramolecular polymers. The ammonium salt byproduct is molecularly dissolved at low concentrations (beige), interacts with BTA monomers at intermediate concentrations (green) and precipitates at high concentrations (blue). b) Simulated CD traces at different concentrations corresponding to a, monitoring the BTA polymers present in solution. Adapted with permission from Ref.[51], © 2022, Wiley‐VCH GmbH.

We have experimentally studied the kinetically controlled assembly of BTA in organic solvents synthesized in situ (Figure 5).^[^ ^51^ ^]^ By substituting the BTA core with the side chains under assembly conditions of BTA, the covalent and non‐covalent reactions could be combined in one experiment. The formation of BTA polymers was then monitored using CD spectroscopy, and reproduced in simulations with a kinetic model. Unexpectedly, the ammonium salt byproduct of the substitution reaction showed an interaction with the product BTA, causing the disassembly of BTA polymers specifically at intermediate concentrations. Such emergent complexity is common in out‐of‐equilibrium polymerizations where multiple components play active roles as cofactors or additives. This type of systems introduces the field of systems chemistry, which focuses on understanding and designing chemical systems with dynamic, self‐regulating behavior, often involving feedback mechanisms and intricate interactions between different molecular species.^[^ ^12^, ^52^ ^]^

## 4 Multicomponent Supramolecular Systems

**Figure 6:**
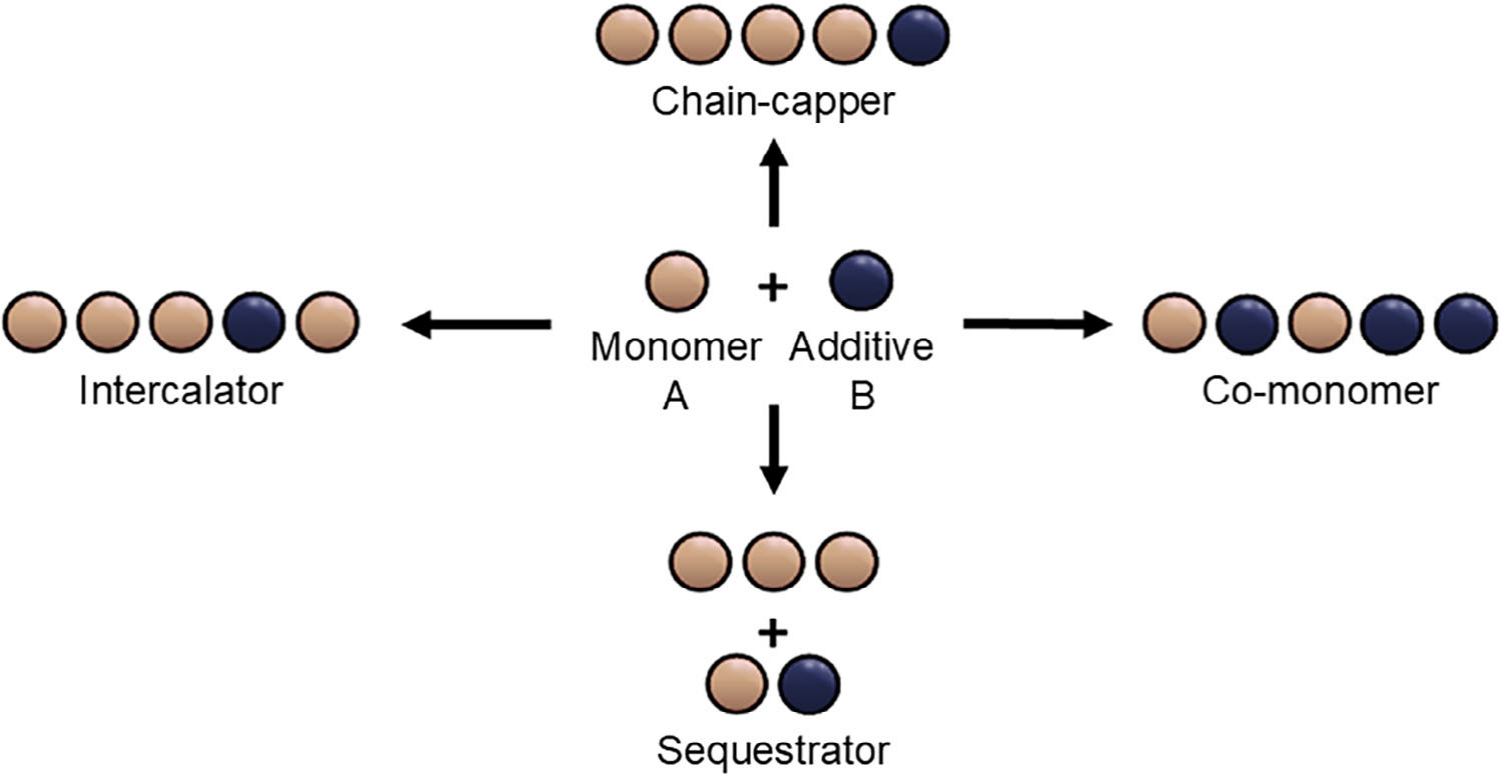
Schematic overview of interaction mode classification for multicomponent supramolecular systems. When added to a monomer, an additive can be 1) a sequestrator, interacting with free monomers, 2) a chain‐capper, interacting with polymer chain ends, 3) an intercalator, mixing in between monomers in the polymers, or 4) a co‐monomer, copolymerizing with the monomers. Adapted with permission from Ref.[54], © 2020, Thieme.

The mixing of multiple components has led to emergent properties, prompting the supramolecular polymer community to take steps toward multicomponent supramolecular systems. However, the diversity of interacting components also introduces complexity in the number of different interaction types.^[^ ^53^ ^]^ Our group has proposed a classification for these additives based on their effect on the primary monomers (Figure 6).^[^ ^54^ ^]^ Specifically, an additive can act as 1) a sequestrator, 2) a chain‐capper, 3) an intercalator, or 4) a co‐monomer.

Sequestrators stabilize monomers in their monomeric state, reducing the availability of free monomers to participate in the self‐assembly process, thereby shifting the equilibrium without affecting the inherent stability of the polymers. Sequestrators are particularly useful in cases where monomer self‐assembly occurs through a cooperative mechanism, as polymerization in such systems is highly sensitive to monomer concentration.^[^ ^55^ ^]^ This effect is similar to the addition of a good solvent to a supramolecular polymer solution, where monomer stabilization drives partial disassembly of the polymer by shifting the equilibrium toward the monomeric state.^[^ ^56^ ^]^ The use of sequestrators has facilitated the development of dilution‐induced supramolecular polymerization systems, where the monomers are sequestered at high concentrations, but released upon dilution.^[^ ^55^, ^57^ ^]^

Chain‐cappers bind non‐covalently to supramolecular polymers, inhibiting monomer addition and chain elongation without altering the structure of the polymer.^[^ ^58^ ^]^ Chain‐cappers are commonly employed to exert thermodynamic control over polymer length; even small amounts can significantly reduce the average degree of polymerization in cooperative systems.^[^ ^59^ ^]^

Intercalators engage in two‐way interactions with monomers, allowing intercalation into the supramolecular polymer, in many ways comparable to host‐guest interactions.^[^ ^60^, ^61^ ^]^ Like typical guests in these systems, intercalators are usually added in small amounts relative to the monomers and modulate the structural and functional properties of the polymer.

Co‐monomers are able to polymerize with the first component and also have sufficient homo‐interactions to aggregate. Fundamental studies, many supported by theoretical modeling, have revealed how the organization within supramolecular aggregates is influenced by the relative strengths of hetero‐ and homo‐interactions.^[^ ^62^, ^63^, ^64^, ^65^, ^66^ ^]^ The ratio of these interactions critically determines the thermodynamically controlled composition of the resulting copolymer, providing insight into the principles of supramolecular copolymerization.^[^ ^67^ ^]^ Current theoretical models offer an initial framework for linking the molecular structures of co‐monomers to the resulting supramolecular architectures. The major current challenge in the field is to translate these theoretical insights into novel molecular systems and functional materials.

**Simulating Assembly Landscapes of Supramolecular Polymer Systems**

**Figure 7:**
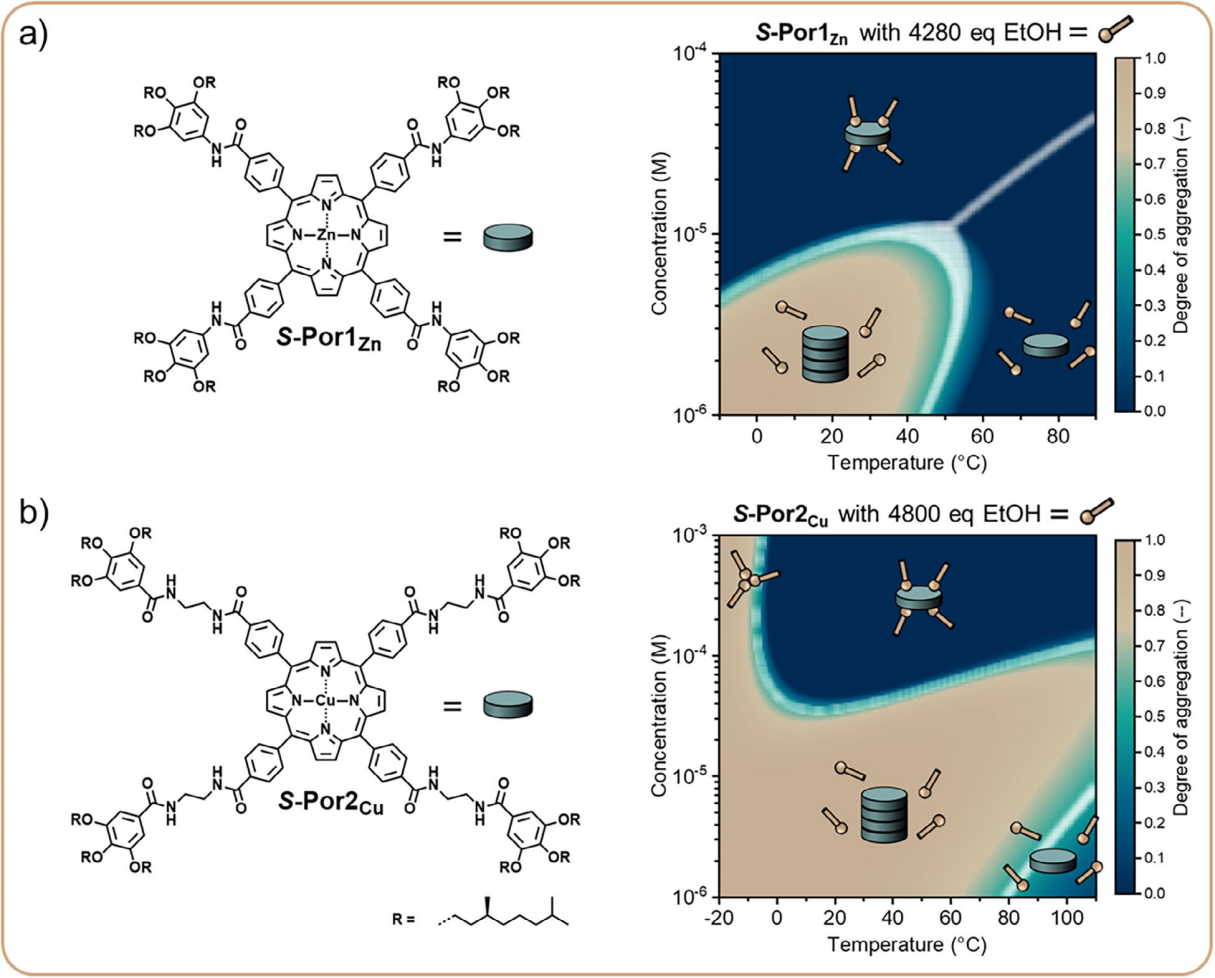
Molecular structures and simulated assembly landscapes of a) S‐Por1_Zn_ with 4280 equivalents ethanol in methylcyclohexane and b) S‐Por2_Cu_ with 4800 equivalents ethanol in methylcyclohexane/chloroform (98/2 v/v). The assembly landscapes are shown as a function of the porphyrin monomer concentration and the temperature. Both monomers showed a preferred interaction with ethanol at high concentrations, facilitating dilution‐induced assembly, and in specific temperature ranges, facilitating thermally bisignate polymerization for S‐Por2_Cu_. Adapted with permission from Ref.[68], © 2023, American Chemical Society.

Together with the group of Prof. Aida, we have recently used computational models to simulate the assembly landscapes of multicomponent supramolecular systems.^[^ ^68^ ^]^ By fitting these models to experimental data of the interactions between porphyrin monomers and alcohol additives in apolar solvents, we uncovered how alcohols act as sequestrators and influence the self‐assembly process under different conditions (Figure 7). The simulated assembly landscape revealed that dilution‐induced supramolecular polymerization^[^ ^55^ ^]^ and thermally bisignate polymerization^[^ ^69^ ^]^ are caused by the same underlying interactions. While our studies examined sequestrators, the methodologies developed can be generalized to analyze other types of additives in multicomponent systems. These findings provide a comprehensive understanding of supramolecular systems and enable the predictive design of systems with desired assembly properties.

**Figure 8:**
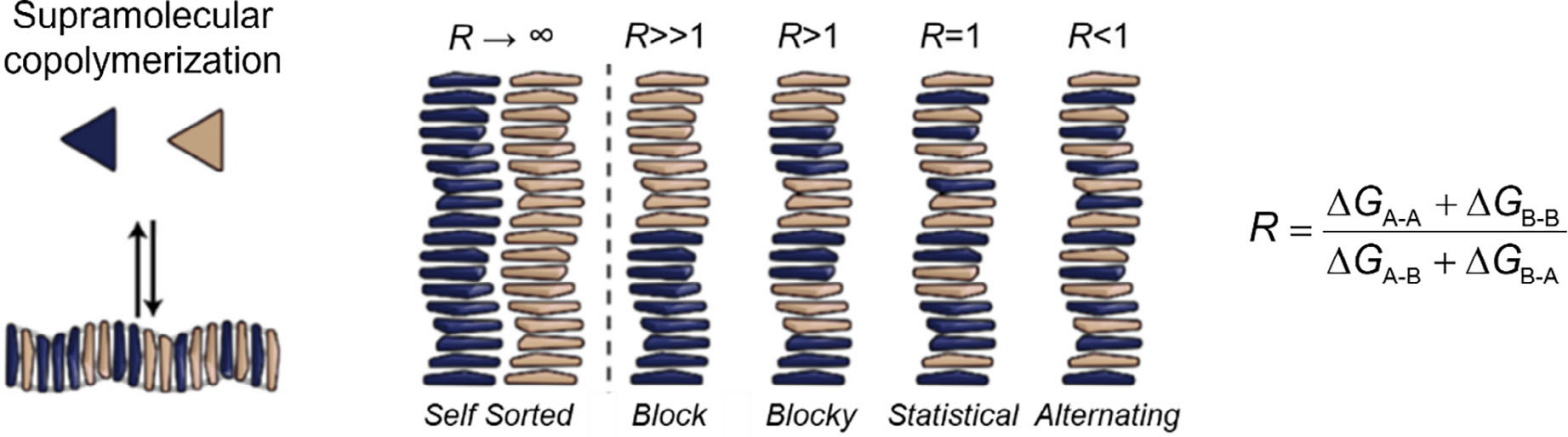
Schematic representation of supramolecular copolymerization and the microstructures that can be formed. The copolymer microstructure depends on the reactivity ratio R, which can be calculated from the relative interaction strength of the homo‐ and hetero‐interactions of the comonomers. Copolymer microstructures can range from block copolymers to alternating copolymers.

The composition of supramolecular copolymers is typically dictated by thermodynamics,^[^ ^70^ ^]^ in contrast to covalent copolymers, where the polymer morphology is often determined by kinetics.^[^ ^71^ ^]^ The supramolecular organization in two‐component materials depends strongly on the strength of the hetero‐interactions relative to the homo‐interactions (Figure 8).^[^ ^65^ ^]^ Strong homo‐interactions and the absence of hetero‐interactions lead to supramolecular self‐sorting of the two components into homopolymers,^[^ ^72^, ^73^, ^74^ ^]^ which could even be used to isolate enantiomers starting from an optically impure compound.^[^ ^75^ ^]^ Self‐sorting has also been observed when two monomers are mixed that pack differently into their respective homopolymers.^[^ ^76^ ^]^ A slight occurrence of hetero‐interactions, for example by smaller conformational differences of the comonomers, can induce mixing of the two compounds into block copolymers.^[^ ^67^ ^]^ In thermodynamically controlled block copolymers, the strength of hetero‐interactions is negatively correlated with the size of the poly(A) and poly(B) blocks. Statistical or random mixing is achieved when there is no penalty or advantage for hetero‐interactions compared to either of the homo‐interactions.^[^ ^66^, ^77^ ^]^ Commonly studied statistically mixed copolymers utilize a second component that is similar to the first component, but functionalized at the sites that are not involved in the supramolecular assembly process.^[^ ^78^, ^79^ ^]^

In contrast, when hetero‐interactions dominate supramolecular aggregation, mixing is advantageous and facilitates an alternating structure of A and B monomers.^[^ ^80^ ^]^ Alternating structures are typically formed by homoditopic dimers,^[^ ^81^ ^]^ heteroditopic dimers,^[^ ^82^, ^83^, ^84^ ^]^ or complementary hydrogen bonding^[^ ^85^, ^86^, ^87^ ^]^ and other hierarchical interactions,^[^ ^88^ ^]^ in which the hetero‐interactions are very specific and the homo‐interactions are attenuated. These classifications help to understand and compare different supramolecular systems.

**Figure 9:**
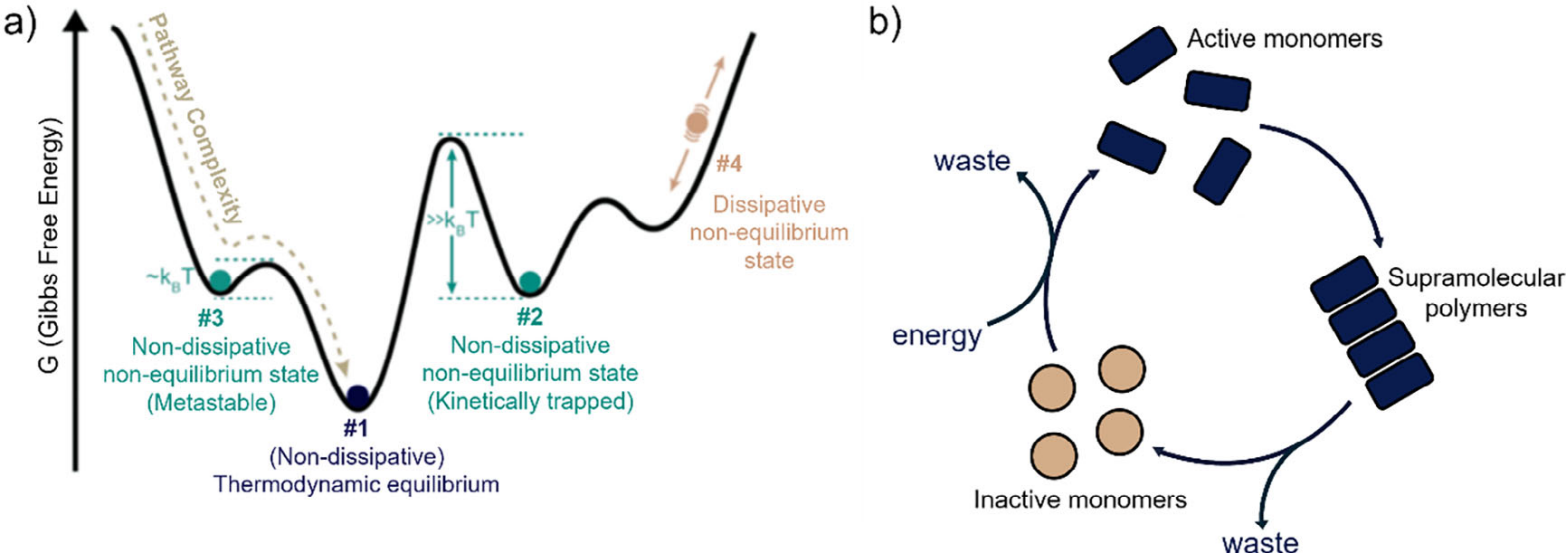
a) Schematic definition of (non‐)equilibrium and (non‐)dissipative supramolecular systems. Adapted with permission from Ref.[43], © 2017, The Royal Society of Chemistry. b) Typical reaction cycle of dissipative supramolecular systems, where energy is required to maintain the assembly state.

In addition to the above‐mentioned supramolecular systems close to thermodynamic equilibrium, non‐equilibrium systems have received much attention (Figure 9a). In some systems, the additives act as so‐called chemical fuels for the assembly of monomers, resulting in dissipative supramolecular polymers (Figure 9b).^[^ ^89^ ^]^ The continuous dissipation of energy allows the decoupling of mechanical properties and dynamics of the assemblies, and provides access to structures that would otherwise be thermodynamically unstable.^[^ ^90^, ^91^ ^]^ Synthetic dissipative assemblies have recently been successfully developed. Van Esch and co‐workers presented a general approach to obtaining transient hydrogels,^[^ ^92^ ^]^ which has been widely recognized and adopted as the first example.

All of these systems use a batchwise fuel supply, whereas natural systems operate with a continuous energy input. By mimicking this continuous energy supply with flow chemistry, synthetic supramolecular systems with oscillating compositions and structures have been developed.^[^ ^93^ ^]^ The development of these supramolecular oscillators has been aided by kinetic models of supramolecular polymerization.^[^ ^94^, ^95^ ^]^ These examples highlight that the properties of multicomponent systems are governed not only by thermodynamic stability but also by kinetic factors, forming structures that are highly sensitive to external stimuli.^[^ ^96^ ^]^ As a result, supramolecular systems can adapt, reorganize, and even evolve, making them attractive candidates for applications ranging from biomaterials^[^ ^97^, ^98^ ^]^ to opto‐electronics.^[^ ^99^ ^]^ However, as the complexity of multicomponent systems increases, so does the need for innovative and scalable methodologies that can efficiently manage and analyze their collective properties.

## 5 Toward the Data‐Driven Study of Multicomponent Supramolecular Systems

The study of multicomponent supramolecular systems presents unique challenges due to their inherent complexity and the large variety of interactions between the components involved.^[^ ^53^ ^]^ While the concept of high‐throughput experimentation has revolutionized many areas of chemical and materials research by enabling rapid screening of large combinatorial spaces,^[^ ^100^, ^101^, ^102^, ^103^, ^104^, ^105^, ^106^ ^]^ its applicability in the field of supramolecular systems has been limited.^[^ ^107^, ^108^, ^109^ ^]^ This is largely due to the intricate nature of sample preparation, which requires precise control over factors such as concentration, stoichiometry, solvent conditions, and temperature.^[^ ^11^, ^110^ ^]^ These specific requirements make high‐throughput approaches not only difficult to set up, but also prone to variability, reducing their reliability in generating the large data sets necessary for meaningful statistical analyses.^[^ ^111^ ^]^ As a result, traditional experimental methods in supramolecular chemistry tend to focus on individual systems or small subsets of components, resulting in a slow accumulation of data. This pace limits our ability to model and predict the assembly characteristics of more complex multicomponent systems. Without sufficient data, conventional computational models struggle to capture the nuances of these systems, further limiting the potential for predictive studies.^[^ ^112^ ^]^

**Figure 10:**
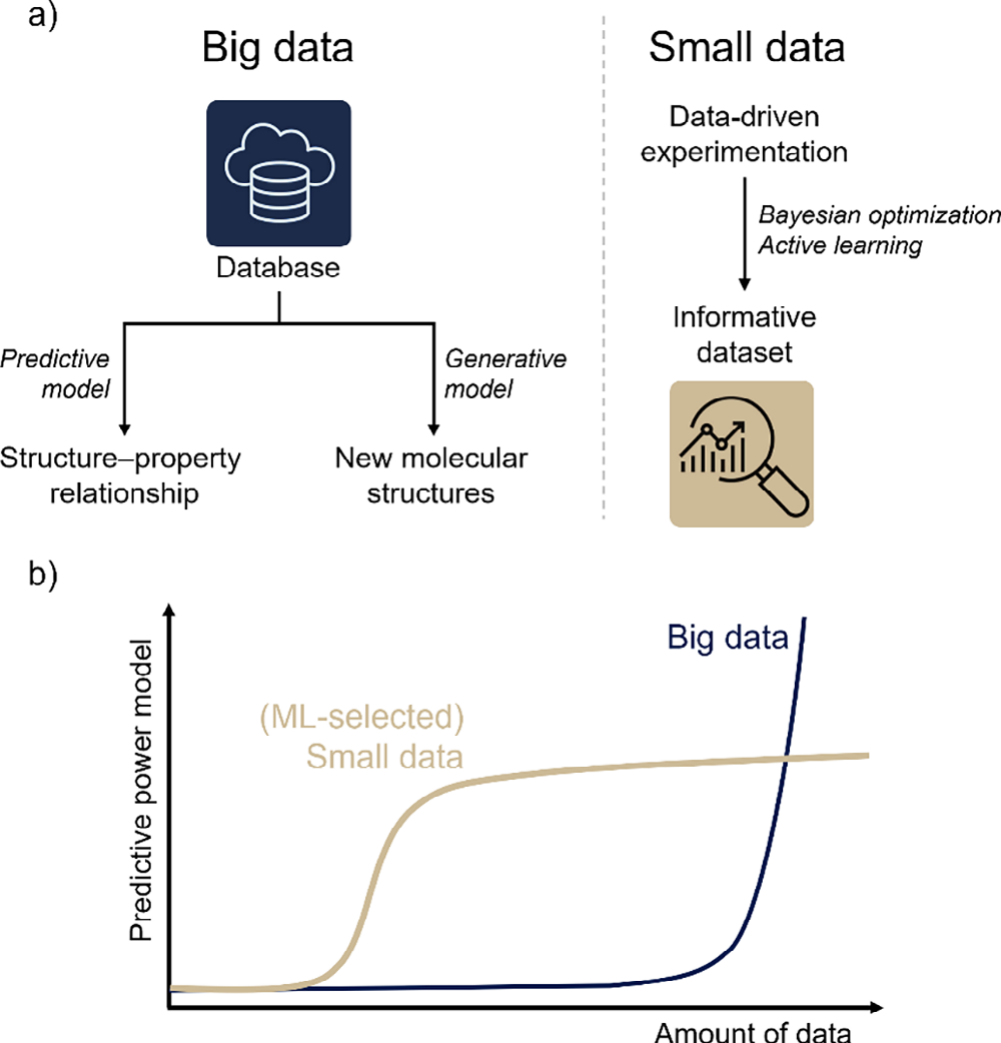
a) Different machine learning approaches based on big data and small data. In big data approaches, large databases can be used to train predictive models that elucidate structure–property relationships, or generative models that suggest new molecules that can lead to improved properties and the discovery of new classes of molecules. Small data approaches often rely on data‐driven experimentation, where data collection is guided by machine learning to acquire the most informative data set that informs and optimizes (a subset of) experimental systems. b) Schematic illustration of the predictive power of machine learning models as a function of the amount of data. Big data approaches require large databases to find trends, but this can lead to generality and high predictive power for large data sets. Small data approaches require less data to make accurate predictions, but these models optimize (a subset of) systems rather than toward generality, leading to a plateau in predictive power.

In contrast, fields such as organic chemistry and drug discovery, have embraced data‐driven approaches, particularly ML, to accelerate research and uncover patterns that may not be immediately apparent using traditional methods (Figure 10a).^[^ ^113^, ^114^, ^115^ ^]^ Vast data sets such as the Protein Data Bank have been instrumental in advancing protein folding predictions^[^ ^116^ ^]^ and enabling de novo protein design,^[^ ^117^ ^]^ which were recognized with the 2024 Nobel Prize in Chemistry. The ability of ML to interpret high‐dimensional, complex data has also driven advances in materials discovery, underscoring its wide‐ranging applicability.^[^ ^118^ ^]^ Despite these successes, the application of ML to multicomponent supramolecular systems remains underexplored, largely because these systems lack the extensive data sets required to develop accurate and generalizable ML models.

However, this gap also highlights a significant opportunity for the application of ML techniques that are well suited for working in the low‐data regime (Figure 10a).^[^ ^119^, ^120^, ^121^ ^]^ In particular, Bayesian optimization (BO) and active learning (AL) stand out as particularly promising approaches for studying supramolecular systems, where data collection is expensive and time‐consuming. BO is an iterative technique that excels at optimizing functions that are expensive to evaluate, such as those found in experimental chemistry.^[^ ^122^ ^]^ By focusing on exploring the most promising regions of a chemical space, BO makes it possible to find optimal or near‐optimal conditions with far fewer experimental trials than traditional methods. Similarly, AL provides a powerful strategy for reducing the number of experiments required to build accurate predictive models.^[^ ^123^ ^]^ In AL, the ML model identifies which data points are most informative for improving its predictions and then prioritizes those data points in subsequent experiments. These iterative methods are universally applicable, and well suited for working in data‐scarce environments. As a result, they have been successfully employed in molecular design,^[^ ^124^ ^]^ material design,^[^ ^125^, ^126^, ^127^, ^128^ ^]^ and reaction optimization.^[^ ^129^, ^130^, ^131^, ^132^ ^]^ Therefore, BO and AL hold great potential for use in supramolecular chemistry, where the cost and difficulty of preparing and testing each sample is a significant barrier (Figure 10b). The application of these data‐efficient ML techniques to supramolecular chemistry could dramatically enhance our ability to study multicomponent systems, leading not only to more efficient experimentation, but also to new discoveries that would be difficult to achieve using traditional methods.^[^ ^133^ ^]^

**Active Learning to Map Assembly Landscapes of Supramolecular Polymer Systems**

**Figure 11:**
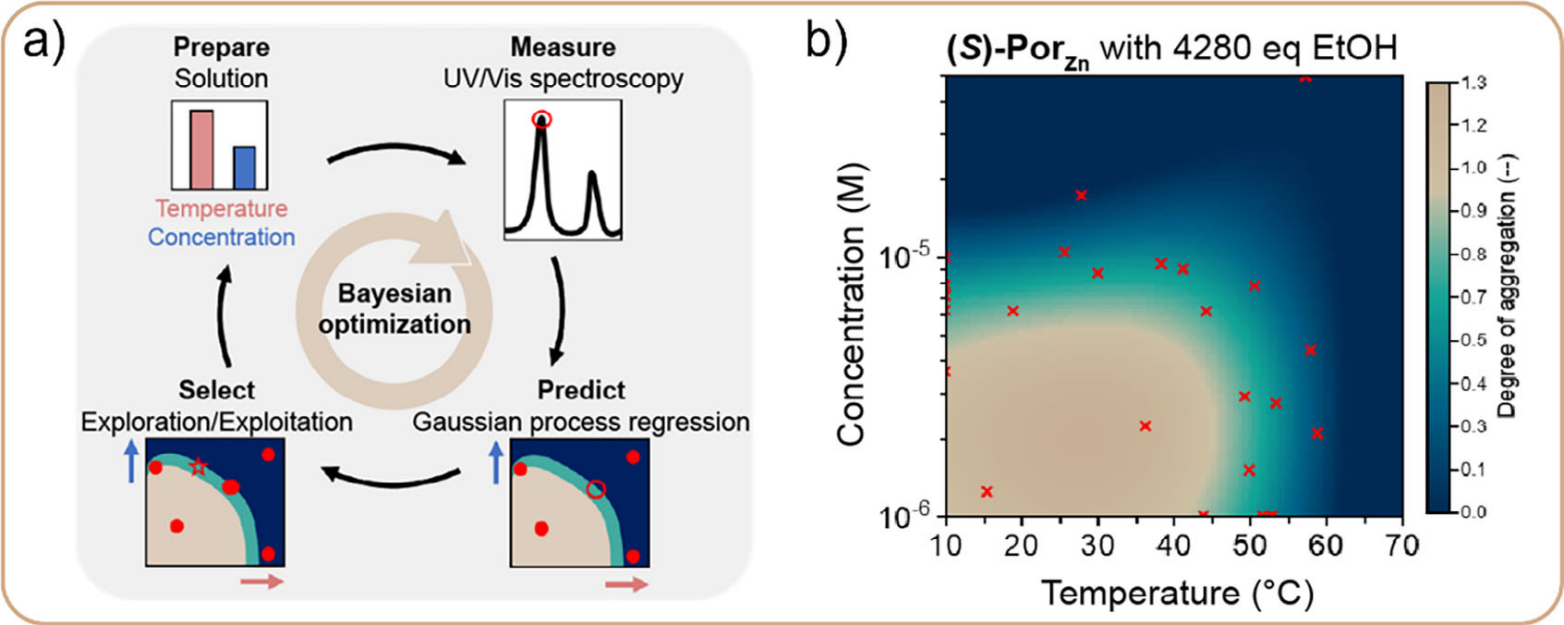
a) Overview of workflow for accelerated mapping of assembly landscapes as a function of temperature and concentration. b) Predicted assembly landscape after 25 experimental iterations, starting with four random experimental data points. Red crosses indicate the experimentally measured data points.

We have recently demonstrated how an AL framework can efficiently explore the complex parameter spaces that govern supramolecular assembly.^[^ ^134^ ^]^ In one of the cases from this work, we used it to experimentally map the assembly landscapes of porphyrin‐based monomers with alcohol additives (Figure 11), extending beyond previous studies that simulated these landscapes. This strategy required no prior knowledge of the experimental system to derive the mass‐balance model, and far fewer experiments than other sampling methods, while still capturing the nonlinear transitions. Such approaches are particularly valuable in supramolecular polymer science, where high‐dimensional design spaces and subtle component interactions are the norm.

## 6 Summary and Outlook

In summary, the convergence of insights from biological protein assemblies and synthetic supramolecular polymers has opened new avenues in the fundamental study of these systems. Recent advances in experimental and computational techniques have not only deepened our mechanistic understanding of self‐assembly but have also revealed the intricate behaviors inherent in multicomponent systems. Despite this progress, the field faces significant challenges related to system complexity and sensitivity, often relying on heuristic approaches and extensive trial‐and‐error efforts.

Looking forward, the integration of ML tools represents a promising strategy to improve the optimization and discovery of supramolecular systems. By using data‐driven models alongside traditional experimental and computational techniques, researchers can achieve more predictive control over assembly processes and material properties. Given the rapidly evolving landscape of ML, we expect that data‐driven approaches will become increasingly prevalent in the study of supramolecular polymers. A wide range of techniques—from BO to various forms of ML—offer new ways to model, explore, and predict complex assembly behavior. These methods differ in how they handle data, make predictions, and scale with system complexity, which becomes particularly relevant in systems that involve distinct monomer types or far‐from‐equilibrium conditions. We encourage future efforts to explore a broader range of methods and work toward a practical guide that can support researchers in choosing suitable data‐driven strategies for different types of supramolecular problems. We advocate a more interdisciplinary approach that brings together chemistry, biology, materials science, and computer science to address these challenges. Such collaborations will be key to unlocking the full potential of supramolecular polymers and translating fundamental insights into practical applications, ultimately driving both therapeutic and technological innovation.

## Conflict of Interests

The authors declare no conflict of interest.

## Data Availability

Data sharing is not applicable to this article as no new data were created or analyzed in this study.
